# Supplementary material for: The Novel Phosphatidylinositol-3-Kinase (PI3K) Inhibitor Alpelisib Effectively Inhibits Growth of PTEN-Haploinsufficient Lipoma Cells
Source: Cancers (Basel). 2019 Oct 17;11(10):1586. doi: 10.3390/cancers11101586 (PMC6826943; doi:10.3390/cancers11101586)
Supplement: Supplementary file 1 [file cancers-11-01586-s001.zip › Alpelisib Cancers Supplement Figures.docx]

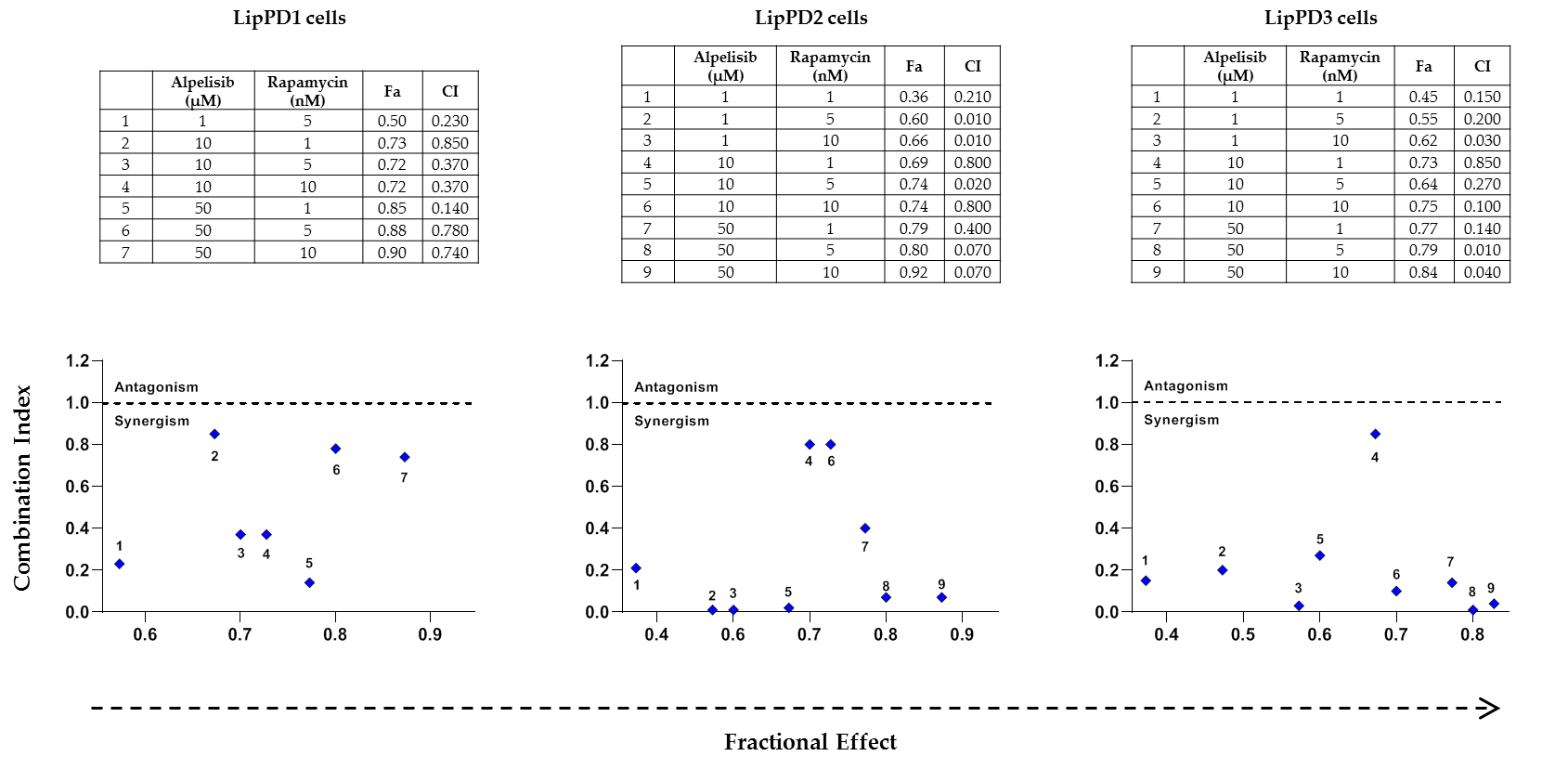


**Figure S1.** Combination index for alpelisib and rapamycin. LipPD1, LipPD2, and LipPD3 cells were treated with alpelisib, rapamycin, or their combination for 72h; and then assessed for cell viability using WST1 assay. Isobologram analysis showed the synergistic anti-tumor activity of alpelisib and rapamycin. The graph (lower panels) is derived from the values given in the table (upper panels). Combination index (CI) <1 indicates synergy. n=3.

|   (**a**) |   (**b**) |
| --- | --- |

**Figure S2.** Immunofluorescence staining of LipPD2 and LipPD3 cells after 72 h of alpelisib treatment: (**a**) Fraction of proliferation marker Ki-67 positive cells was decreased after alpelisib treatment, n=2. (**b**) Fraction of pS6 positive cells was decreased after alpelisib treatment, n=3. Fold over solvent control (black line), * p≤0.05.

| 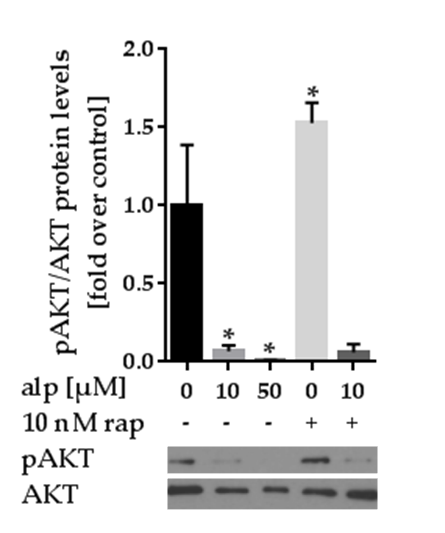  (**a**) | 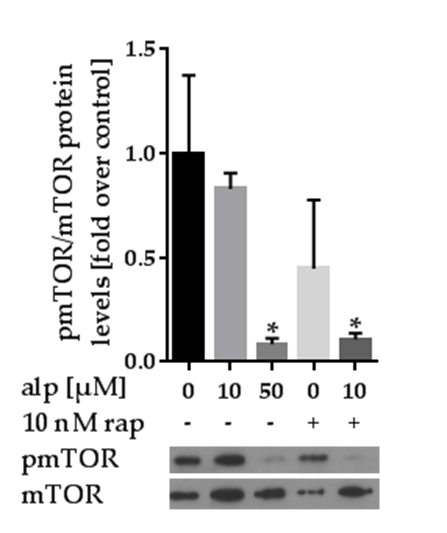  (**b**) | 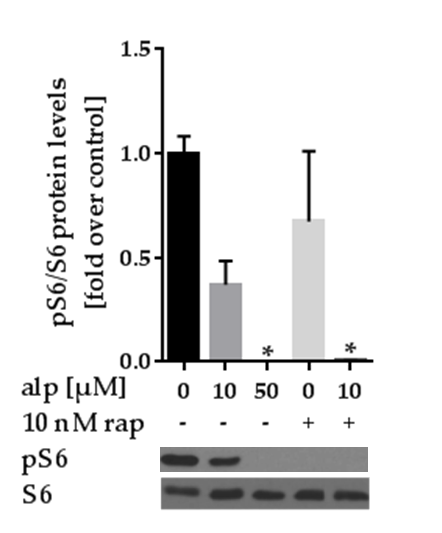  (**c**) |
| --- | --- | --- |

**Figure S3.** Western blot analysis of LipPD2 cells after 24 h treatment with alpelisib (alp) and/or rapamycin (rap), representative blots and densitometric analysis: (**a**) Phosphorylated AKT (phospho-Thr 308 (pAKT) normalized to total AKT) was reduced after alpelisib but elevated during rapamycin treatment; (**b**) phosphorylation of mTOR (phospho-Ser 2448 (pmTOR) normalized to total mTOR) was reduced after 50 µM alpelisib and combined treatment with rapamycin; (**c**) phosphorylation of ribosomal protein S6 (phospho-Ser 235/236 (pS6) normalized to total S6 protein) was reduced after 50 µM alpelisib and combined treatment with rapamycin. One representative blot out of 3 for each protein (phosphorylated and total) and densitometric analysis of three independent experiments is shown, fold over solvent control, * p≤0.05.

| 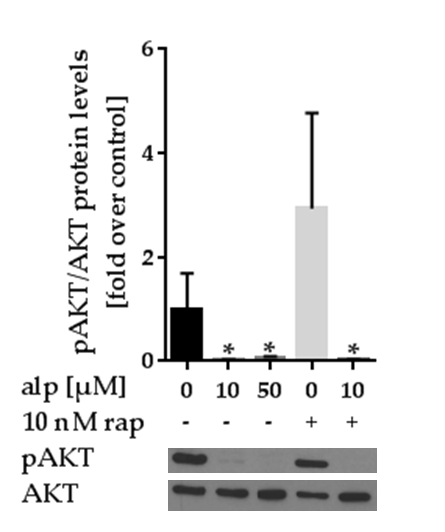  (**a**) | 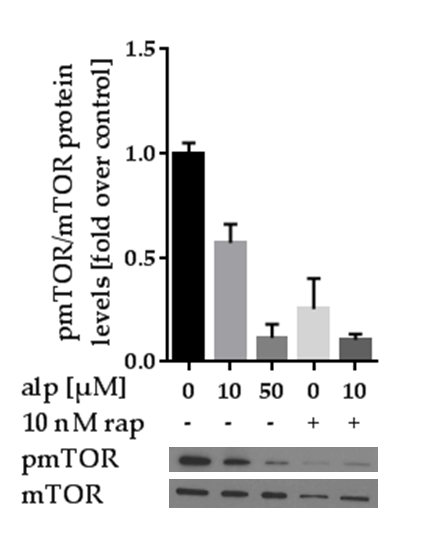  (**b**) | 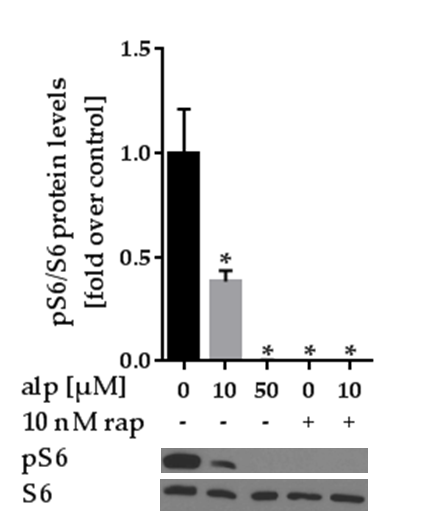  (**c**) |
| --- | --- | --- |

**Figure S4.** Western blot analysis of LipPD3 cells after 24 h treatment with alpelisib (alp) and/or rapamycin (rap), representative blots and densitometric analysis: (**a**) Phosphorylated AKT (phospho-Thr 308 (pAKT) normalized to total AKT) was reduced after alpelisib but not rapamycin treatment; (**b**) phosphorylation of mTOR (phospho-Ser 2448 (pmTOR) normalized to total mTOR); (**c**) phosphorylation of ribosomal protein S6 (phospho-Ser 235/236 (pS6) normalized to total S6 protein) was reduced after alpelisib and rapamycin treatment. One representative blot out of 3 for each protein (phosphorylated and total) and densitometric analysis of three independent experiments are shown, fold over solvent control, * p≤0.05.

Alpelisib [µM] #1 0 10 50 0 10 #2 0 10 50 0 10

Rapamycin 10 nM - - - + + - - - + +

pAKT
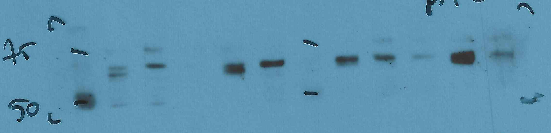


AKT
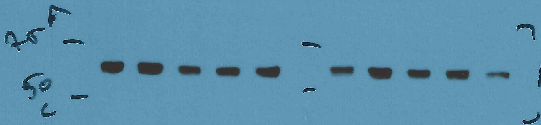


pmTOR
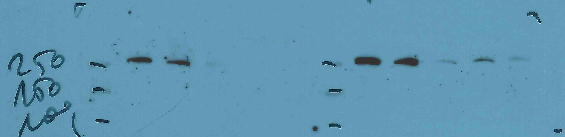


mTOR
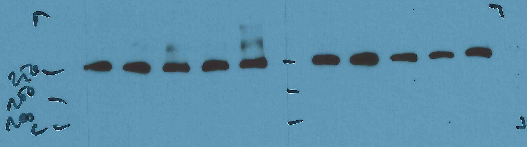


pS6
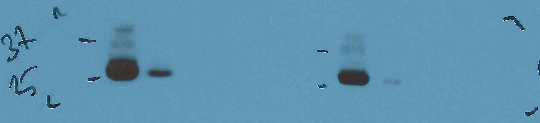


S6
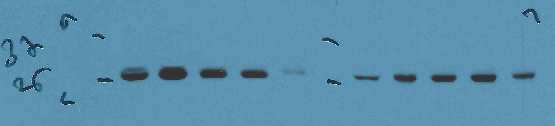


GAPDH
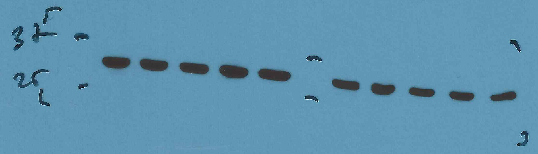


Alpelisib [µM] #3 0 10 50 0 10 0 0 10 50 0

Rapamycin 10 nM - - - + + - - - - + red: repeat of #2, not used

pAKT
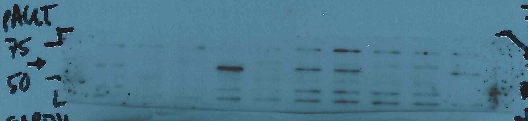


AKT
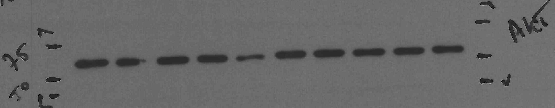


pmTOR
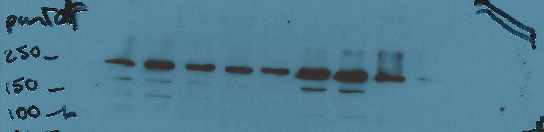


mTOR
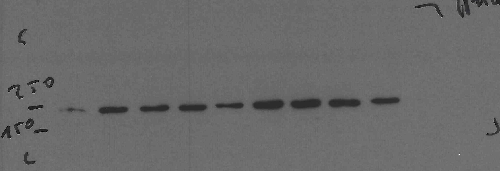


pS6
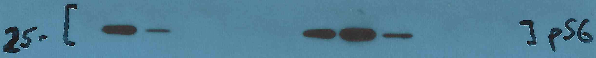


S6
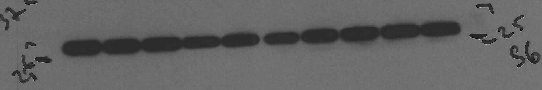


GAPDH
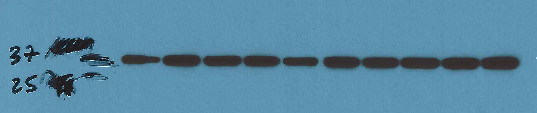


**Figure S5.** Western blots after 24 h treatment with alpelisib and/or rapamycin in LipPD1.

Alpelisib [µM] #2 10 0 50 10 0 #1 10 0 50 10 0 #2: LipPD2 #2, #1: LipPD2 #1

Rapamycin 10 nM + + - - - + + - - -

pAKT
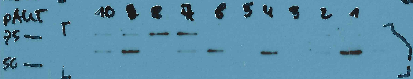


AKT
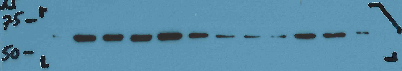


pmTOR
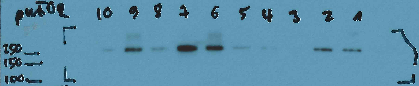


mTOR
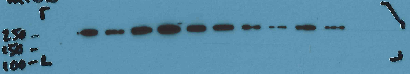


pS6
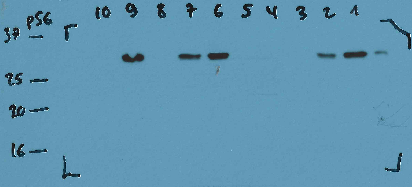


S6
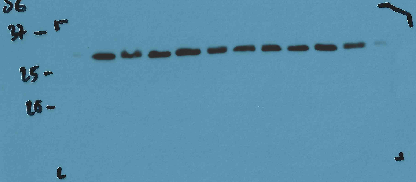


GAPDH
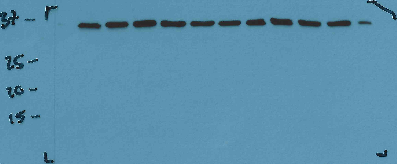


Alpelisib [µM] #1 0 10 50 0 10 #3 10 0 50 0 0 black: LipPD2 #3

Rapamycin 10 nM - - - + + - - - + + red: LipPD3 #1

pAKT
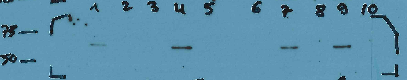


AKT
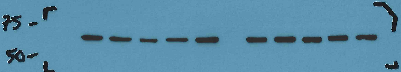


pmTOR
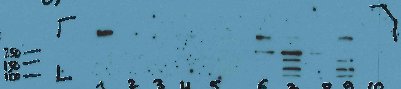


mTOR
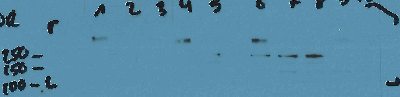


pS6
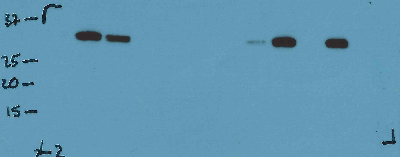


S6
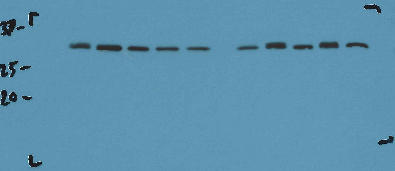


GAPDH
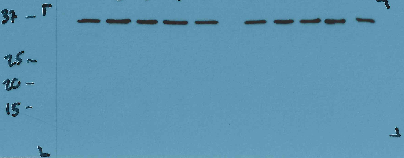


**Figure S6.** Western blots after 24 h treatment with alpelisib and/or rapamycin in LipPD2 and LipPD3 #1.

Alpelisib [µM] #2 0 10 50 0 10 #3 0 10 50 10 0 #2: LipPD3 #3, #3: LipPD3 #3

Rapamycin 10 nM - - - + + - - - + +

pAKT
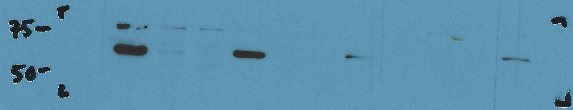


AKT
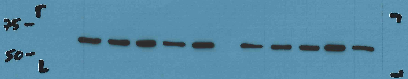


pmTOR
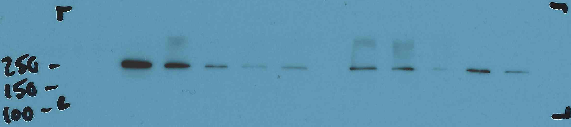


mTOR
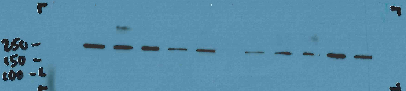


pS6
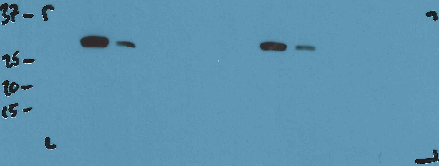


S6
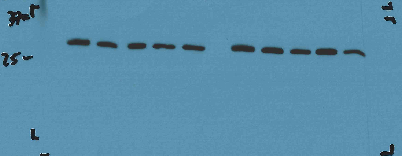


GAPDH
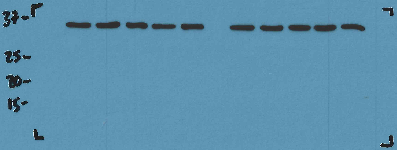


**Figure S7.** Western blots after 24 h treatment with alpelisib and/or rapamycin in LipPD3 (#2 and #3).

|  |
| --- |

**Figure S8.** RT-qPCR of *Fatty acid synthase* (FASN) gene from LipPD1 cells after 10 days of differentiation with or without 10 µM alpelisib: mRNA expression of *FASN* was downregulated in 10 µM alpelisib-treated cells. Fold over control, values were normalized to the housekeeper gene *β-actin*, n=3 p=0.064.

|   (**a**) |   (**b**) |
| --- | --- |

**Figure S9.** β-galactosidase senescence staining of lipoma cells after 72 h alpelisib treatment: (**a**) Fraction of senescent LipPD2 cells increased with 10 µM alpelisib compared to solvent control, n=2, p=0.236 ; (**b**) Fraction of senescent LipPD3 cells increased with 10 µM alpelisib compared to solvent control, n=3, p=0.065.

|   (**a**) |   (**b**) |
| --- | --- |
|  (**c**) |  (**d**) |

**Figure S10.** RT-qPCR of senescence, stem cell and adipogenesis marker genes from LipPD1-3 cells before or after 10 days of differentiation with or without 10 µM alpelisib: mRNA expression of (**a**) *p16* was increased (p=0.222), (**b**) *CD44* was increased (p=0.015) and (**c**) *CD90* was decreased in 10 µM alpelisib-treated cells (p=0.41). Fold over control, values were normalized to the housekeeper gene *HPRT*, n=3, * p≤0.05 determined via paired *t*-test.
